# Bioregional Alterations in Gut Microbiome Contribute to the Plasma Metabolomic Changes in Pigs Fed with Inulin

**DOI:** 10.3390/microorganisms8010111

**Published:** 2020-01-13

**Authors:** Weida Wu, Li Zhang, Bing Xia, Shanlong Tang, Lei Liu, Jingjing Xie, Hongfu Zhang

**Affiliations:** 1 State Key Laboratory of Animal Nutrition, Institute of Animal Sciences, Chinese Academy of Agricultural Sciences, Beijing 100193, China; harrypolowwd87@163.com (W.W.); xiabingcaas@126.com (B.X.); long18763897938@163.com (S.T.); swina2010@163.com (L.L.); zhanghongfu@caas.cn (H.Z.); 2 State Key Laboratory of Food Science and Technology, School of Food Science and Technology, Nanchang University, No. 235, East Nanjing Road, Qingshan Lake District, Nanchang 330047, China; douweibahua@163.com

**Keywords:** inulin, microbiota, BCAA, IPA, multi-omics

## Abstract

Inulin (INU) is a non-digestible carbohydrate, known for its beneficial properties in metabolic disorders. However, whether and how gut microbiota in its regulation contributes to host metabolism has yet to be investigated. We conduct this study to examine the possible associations between the gut microbiota and circulating gut microbiota–host co-metabolites induced by inulin interventions. Plasma and intestinal site samples were collected from the pigs that have consumed inulin diet for 60 days. High-throughput sequencing was adopted for microbial composition, and the GC-TOF-MS-based metabolomics were used to characterize featured plasma metabolites upon inulin intervention. Integrated multi-omics analyses were carried out to establish microbiota–host interaction. Inulin consumption decreased the total cholesterol (*p* = 0.04) and glucose (*p* = 0.03) level in serum. Greater β-diversity was observed in the cecum and colon of inulin-fed versus that of control-fed pigs (*p* < 0.05). No differences were observed in the ileum. In the cecum, 18 genera were altered by inulin, followed by 17 in the colon and 6 in the ileum. Inulin increased propionate, and isobutyrate concentrations but decreased the ratio of acetate to propionate in the cecum, and increased total short fatty acids, valerate, and isobutyrate concentrations in the colon. Metabolomic analysis reveals that indole-3-propionic acid (IPA) was significantly higher, and the branched-chain amino acids (BCAA), L-valine, L-isoleucine, and L-leucine are significantly lower in the inulin groups. Mantel test and integrative analysis revealed associations between plasma metabolites (e.g., IPA, BCAA, L-tryptophan) and inulin-responsive cecal microbial genera. These results indicate that the inulin has regional effects on the intestine microbiome in pigs, with the most pronounced effects occurring in the cecum. Moreover, cecum microbiota plays a pivotal role in the modulation of circulating host metabolites upon inulin intervention

## 1. Introduction

A plethora of studies suggest that the dietary fibers, such as inulin, confer health benefits to host metabolism; protect against the metabolic disorder; and reduces the risk of inflammatory bowel diseases [1], cardiovascular diseases [2], obesity [3], and type 2 diabetes mellitus (T2D) [4] in human. Inulin encompasses all β (2→1) linear fructans of varying chain lengths. It has generally been recognized as safety status in many countries and is extensively employed in formula foods. Inulin influences directly on the gut, including prebiotic effects, improvement of bowel function and glycolipid metabolism in the host, secretion of satiety hormone and increased short fatty acids (SCFAs) production [5,6,7,8]. Although the exact mechanism has not been elucidated, the gut microbiota has been considered as a potential modulating factor [9].

There is considerable site-specificity in gut microbiota composition, with diverse populations residing within each gut location [10,11]. Unique contributions of the gut microbiota at each site to overall host health are not yet fully clarified but are likely dependent on diet ingredients and overall health of the host [12]. The gut microbiome in combination with diet, with the subsequent translocation of microbial metabolites from the intestine to the bloodstream, is likely to play a pivotal role in influencing systemic metabolism [13,14,15]. Inulin-associated alterations in the composition of the gut microbiota are well documented. Inulin promotes the growth of genera *Prevotella, Blautia, Veillonella, and Faecalibacterium* [16,17,18], which produced more SCFAs.

Although SCFAs are the most well-known gut-derived metabolite that affects host metabolism, they are not the sole regulators of host physiology. Changes in the gut microbiome lead to alterations in many microbe-derived metabolites, termed ‘‘xeno-metabolites,’’ that also likely affect host physiology [19]. Interestingly, we had found that inulin could also affect amino acid, especially branch chain amino acids (BCAA) concentration in plasma, which concurs with other studies [20,21,22]. BCAA are essential amino acids and must be obtained from the diet. BCAA not only act as building blocks for tissue protein but also have other metabolic functions [23]. As a biomarker for the early pathogenesis of metabolic diseases, elevation of BCAA is attributed to an increased potential for BCAA biosynthesis and reduced potential for BCAA transport into bacterial cells, the gut microbiota has a major part to manage the BCAA metabolism [24,25]. However, it is still not known whether the BCAA alteration is caused by the microbiota upon inulin intervention and, thus, may be an intrinsic pathway influencing the host metabolism.

Circulating metabolite levels often act as intermediaries between states of the gut microbiota and host biology [26,27]. Therefore, integrated study of microbiome and metabolome has been suggested as the most promising approach to evaluate host–microbiome interactions [28]. Besides, the gastrointestinal tract of pigs is known to share characteristics with the structure of humans, and similar metabolic profiles were observed when pigs and humans consumed the same dietary fiber. Thus, the pig model provides a useful framework for identifying dietary-related alteration in host–microbiome interactions [29]. To address the question whether the gut microbiome mediates the regulatory effect of inulin on the circulating metabolome, we used a 16S rRNA sequencing and metabolome integrated multi-omics approach to explore possible associations among specific bacterial populations, functionalities and circulating gut microbiota-host co-metabolites. These relationships could help decipher potential underlying mechanisms by which how inulin consumption ultimately affects host metabolism.

## 2. Materials and Methods

### 2.1. Experimental Pigs and Ethics Statement

Twelve crossbred (Duroc × Yorkshire) barrows were randomly assigned one of two diets in a randomized design with six animals per treatment. Pigs were individually fed and housed in metabolism pens (1.2 m × 1.5 m) with feed and water trough. Lights on from 7:00 a.m. to 8:00 p.m., and experimental animal rooms were kept 22 ± 2 °C through the experimental period. Pigs fed *ad libitum* and had free access to water. Each group of pigs was fed with a maize-soybean meal diet containing 5% a high-fermentable fiber, (inulin, Vilof Agricultural Technology Co., Hebei, China) and 5% a low-fermentable fiber control (cellulose, microcrystalline cellulose, CON, Beijing NCC Technology R&D Center, Beijing, China) respectively. Cellulose was used as a control attributed to its low fermentability and consequent low SCFAs in hindgut. All experiment diets were formulated isocalorically and isonitrogenously (Table S1) to meet or exceed the nutritional requirements of the corresponding physiological period suggested by NRC (2012). Experimental animals were healthy and did not receive any antibiotic treatment within the experimental period. All experimental procedures were approved by the Animal Welfare Committee in the Institutes of Animal Sciences, Chinese Academy of Agricultural Sciences on May 4, 2017 (Ethics Code Permit IAS2017-3), and were compliant to the Regulations for the Administration of Affairs Concerning Experimental Animals (The State Science and Technology Commission of P. R. China, 1988).

### 2.2. Blood Samplings

Before the last morning meal, blood samples were drawn from the front cavity vein, then kept on ice. Plasma and serum were separated by centrifugation for 10 min at 3000 *g* at 4 °C and stored in aliquots at −80 °C. Plasma samples were used for metabolomics analyses, while serum samples were used to measure cholesterols, triglycerides (TG), glucose, high-density lipoprotein cholesterol (HDL-C), and low-density lipoprotein cholesterol (LDL-C).

### 2.3. 16S Ribosomal RNA Amplicon Sequencing

After 60 days, every pig of each treatment was slaughtered, and the ileum cecum and colon were immediately separated. Contents from 15 cm proximal to the ileal-caecal junction, mid-cecum, and proximal colon were collected, kept in sterile tubes (2 mL), and immediately frozen at −80 °C for DNA analysis of the bacterial community and for short-chain fatty acids (SCFAs) concentrations.

Genomic DNA was extracted using the manufacturer’s protocol with the EZNATM Soil DNA kit (D5625-02, Omega Bio-Tek Inc., Norcross, GA, USA). The V3–V4 hypervariable regions of the bacterial 16S rDNA were amplified by PCR using primers 338F (5′-ACTCCTRCGGGAGGCAGCAG-3′) and 806R (5′-GGACTACCVGGGTATCTAAT-3′) with unique 8-bp barcodes to facilitate multiplexing and sequencing were carried out with an Illumina sequencing platform. The clustering of V3-V4 rRNA reads at 97% nucleotide sequence into operational taxonomic units (OTUs) was performed using QIIME software [30]. OTUs that contained 0.01% of total reads were filtered out. The Ribosomal Database Project classifier (RDP, version 11.1, http://rdp.cme.msu.edu/) was used for taxonomic assignment.

### 2.4. SCFA and Untargeted Plasma Metabolomics Analyses

The composition of SCFAs in three gut segments was determined using gas chromatography. Firstly, about 2g wet digesta were thoroughly mixed with 10 ml distilled water to extract SCFA. After incubating at 4 °C for 48h and centrifuging at 2000 *g* for 10 min at 4 °C, metaphosphoric acid (25%, *v*/*v*) was added into the supernatant at a ratio of 1:5 for removing protein. Then, the mixed sample was centrifuged at 9000 *g* for 10 min at 4 °C, each supernatant was subjected for SCFA analysis with Agilent 6890N GC (Agilent Technologies, Inc., Palo Alto, CA, USA). Plasma was subjected to untargeted metabolomics analyses using gas chromatography with a time of flight mass spectrometer (GC-TOF-MS), as described by our previous study [20]. Briefly, Liquid-liquid extraction method was used to extract metabolites. Ribitol was spiked as an internal standard (20 μL stock solution of 20 mg/mL in H_2_O). After extraction and derivatization, all samples were analyzed on the GC-TOF-MS. Raw data were processed using MassLynx software (Waters Co., Milford, MA, USA) for peak discrimination, filtering, and alignment. The metabolites were identified from the NIST library and the Human Metabolome Database (HMDB). (http://www.hmdb.ca/).

### 2.5. Statistical Analyses

#### 2.5.1. Body Weight, SCFAs and Serum Fasting Metabolites

Each pig was utilized as an experimental unit. The data of body weight, digesta SCFAs, and serum fasting metabolites were analyzed by ANOVA using JMP 13.0 (SAS Institute, Inc., Cary, NC, USA). The significance for all tests was considered when *p* < 0.05.

#### 2.5.2. Microbiome

Unless otherwise noted, all statistical analyses of operational taxonomic unit (OTU) reads were performed using R (V3.3.0, https://www.r-project.org/). Samples were rarefied to even sampling depths before computing within-samples compositional diversities (Chao1 and Simpson) and between-samples compositional diversity (Bray-Curtis). 2-factor ANOVA was used to assess group differences in α-diversity measurements for main effects (diet and location) and the interaction term. Diet differences within each intestine location were evaluated by the Kruskal-Wallis test. Non-metric multidimensional scaling (NMDS) based on Bray-Curtis distance matrices was performed to obtain a 2-dimensional representation of the groups. A permutational multivariate ANOVA test was performed on the Bray-Curtis matrices using 999 random permutations at a significance level of 0.05. Pairwise comparisons based on a negative binomial Wald test from the DESeq2 software package (Bioconductor) was used to measure the dietary differences in individual OTUs at different taxon levels [31,32]. A value of *p* at 0.05 was considered statistically significant and was corrected for multiple testing using the BH procedure to control the false-discovery rate [33]. Functional profiles were predicted from obtained 16S rRNA gene data using Tax4Fun [34].

#### 2.5.3. Metabolomics

After Pareto scaling and logarithmic transformation, data were analyzed to perform partial least squares discriminant analysis (PLS-DA) for pattern recognition. The PLS-DA model performance was evaluated by the goodness of predictability parameter (Q2) and ten-fold-cross-validation of the data was conducted. Finally, the sample identifiers were verified with 1000 random permutations. Metabolites of interest were considered only when VIP > 1 in PLS-DA models and *p* < 0.05 in a pairwise comparison performed by the nonparametric test. The pathway enrichment and topological analysis was executed on Metabolic Pathway Analysis 4.0 (MetPA, http://www.metaboanalyst.ca/).

Integrative of multi-omics results were using the “mixOmics” package, and a correlation cut off of 0.60 was considered significant [35]. The relationships between the relative abundances of identified genus and normalized metabolites were established by partial spearman’s correlation coefficients.

## 3. Results

### 3.1. Growth Performance and Serum Metabolites

**Figure 1:**
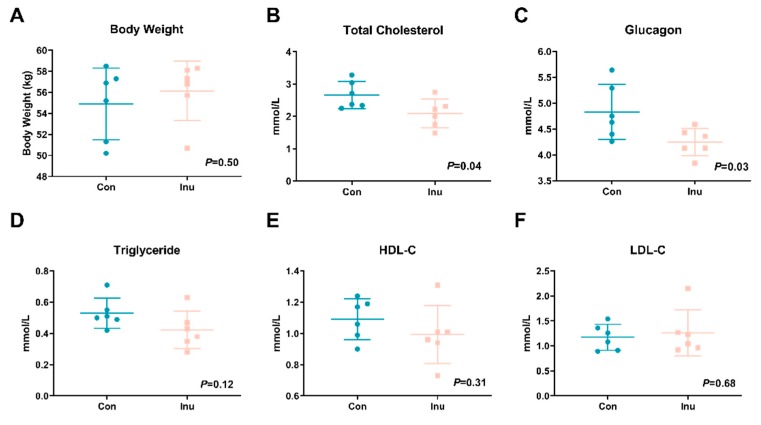
Body weight (**A**), fasting serum total cholesterol (**B**), glucose (**C**), triglyceride (**D**), high-density lipoprotein cholesterol (HDL-C) (**E**) and low-density lipoprotein cholesterol (LDL-C) (**F**) in growing pigs fed a diet with or without a 5% inulin supplement. All data are expressed as mean ± SD (*n* = 6).

Initial body weight was comparable between the two groups. After being fed with the experimental diets, the body weight of pigs was not affected by inulin feeding (*p* = 0.50, Figure 1A). Fasting circulating total cholesterol (*p* = 0.04) and glucose (*p* = 0.03) in the pigs fed with inulin was lower than the control pigs (Figure 1B,C). No difference was revealed in serum triglycerides (*p* = 0.12), HDL-C (*p* = 0.31), and LDL-C (*p* = 0.68).

### 3.2. Inulin has Little Effects on Bacterial α-Diversity, but Change Significantly Hindgut Microbiome

Sample diversity (Shannon index) did not show a significant interaction between inulin and intestinal site at all taxonomic levels. In contrast, sample richness (Chao1) revealed a significant interaction between diet and region except family (Table S2). We analyzed the effect of inulin on α-diversity within intestinal regions (Table S3). No dietary effects were observed for inulin feeding in the ileum. At the phylum, class, order, and family level, inulin resulted in lower sample richness in the cecum relative to con feeding. Only inulin reduced the Shannon index in the cecum at the order level, and in the colon at the family level (Table S4).

**Figure 2:**
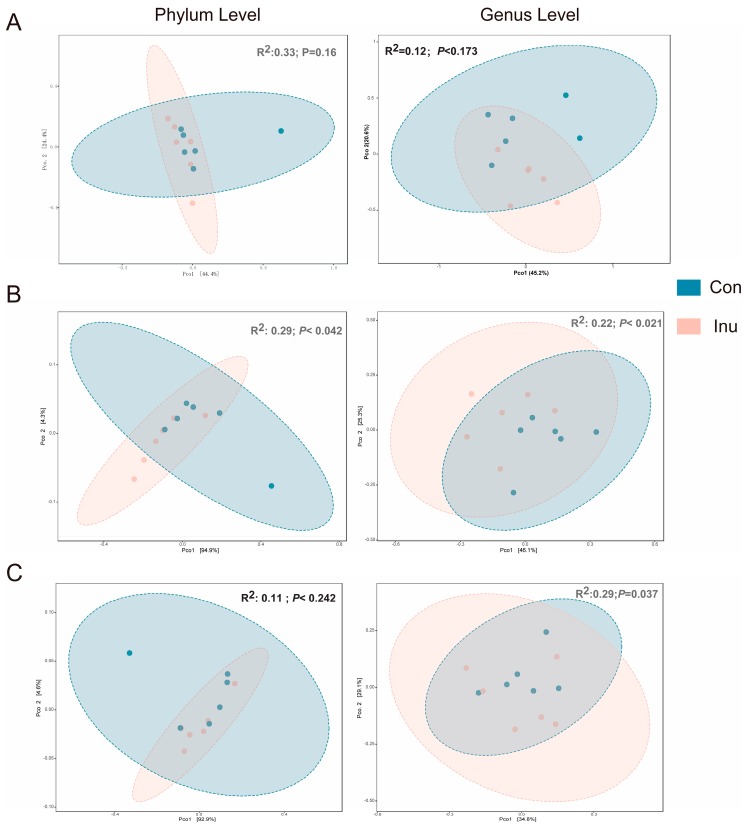
Effect of inulin on bacterial community across luminal regions in growing pigs fed a diet with or without a 5% inulin supplement. Distances created with Bray-Curtis show phylum or genus level in different luminal regions, Ileum (**A**), Cecum (**B**), Colon (**C**). *p* value represents diet differences among PCoA scores along component 1. Con, control; Inu, inulin.

Based on the PERMANOVA analysis on the weighted-Unifrac dissimilarity matrix, we observed that the region-specific β-diversity shifts due to the inulin feeding. Significant diet-specific differences were observed in the cecum and colon at the genus level (cecum *R*^2^ = 0.22, *p* < 0.02; colon *R*^2^ = 0.31, *p* < 0.03), however, dietary discrimination of samples was not as obviously observed in PCoA from ileal samples (Figure 2).

**Figure 3:**
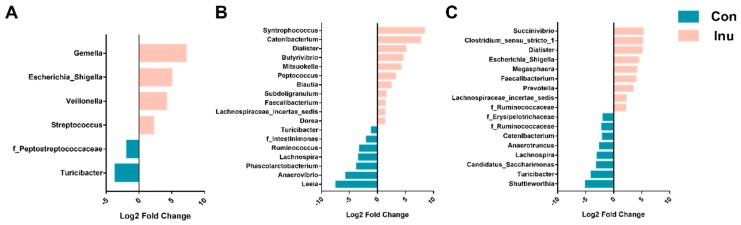
Differentially abundant (*p* < 0.05) genera from the ileum (**A**), cecum (**B**), and colon (**C**) in growing pigs fed a diet with or without a 5% inulin supplement, as determined by DeSeq2. OTUs clustered at 97% similarity were combined by taxonomic classification at the genus level. The results shown are log2 fold change between the CON (control; blue) and INU (inulin; red)-fed groups; note that the x-axis scale is different for each panel. Positive log-fold changes indicate that a genus is enriched in the INU group, while negative log-fold changes indicate that a genus is enriched in the CON group. Con, control; Inu, inulin.

A total of 9 bacterial phyla were observed across all intestine segments. Firmicutes represented the most abundant phylum across all of intestinal regions, with relative abundances of 89.3%, 80.2%, and 53.4% in the ileum, cecum, and colon, respectively. *Bacteroidetes* was highly increased from 1.22% in the ileum to 17.6 and 11.3% in the two regions of the large intestine. It is interesting to note that the ratio of *Firmicutes: Bacteroidetes* was significantly reduced in inulin-fed pigs cecum regions (10.58 vs. 3.78, *p* = 0.05). We further determined significantly altered genera across three regions, 28 genera were significantly different between the diet groups in all intestinal regions (Figure 3). In the ileum region, genus *Gemella*, *Veillonella*, *Escherichia_Shigella*, and *Streptococcus* were more abundant in the inulin group, while *Turicibacter* and an unspecified genus within the *Peptostreptococcaceae* were lower in inulin-compared to con group (Table S5a). Most genera were altered in the cecal region, *Dialister*, *Prevotella*, *Megasphaera*, *Mitsuokella*, *Faecalibacterium*, *Catenibacterium*, *Lachnospiraceae_incertae_sedis*, *Clostridium_sensu_stricto_1*, *Succinivibrio*, and an unassigned genus from *Ruminococcaceae* were significantly more abundant, relative to con group pigs. The genera *Turicibacter*, *Lachnospira*, an unassigned genus from *Ruminococcaceae*, *Shuttleworthia*, *Candidatus_Saccharimonas* and an unassigned genus from *Erysipelotrichaceae* were less abundant in the inulin-fed group compared to con-pigs (Table S5b). In the colon of inulin-fed pigs, *Catenibacterium*, *Syntrophococcus*, *Blautia*, *Dialister*, *Mitsuokella*, *Subdoligranulum*, *Dorea*, *Butyrivibrio*, *Peptococcus*, an unassigned genus from *Lachnospiraceae* and *Faecalibacterium* were more abundant, while *Leeia*, *Ruminococcus*, *Anaerovibrio*, *Phascolarctobacterium*, *Lachnospira*, an unassigned genus within the family *Intestinimonas* and *Turicibacter* were lower than the control group. Additionally, *Turicibacter* was decreased in inulin-fed piglets compared to control-fed piglets, across all three luminal regions. Interestingly, *Faecalibacterium* was found to be greater in the inulin-fed group in the cecal and colonic lumen compared to control-fed piglets (Table S5c).

### 3.3. SCFA Production Was Altered by Dietary Inulin

**Figure 4:**
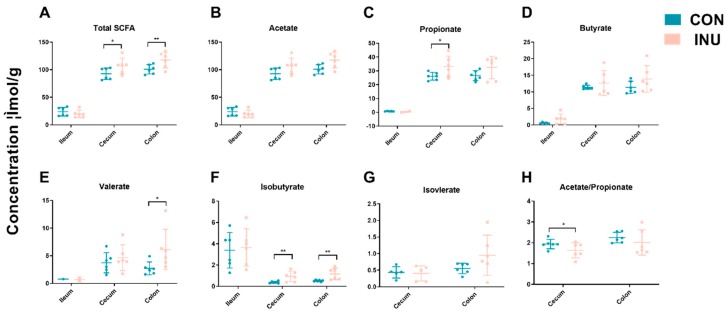
Short chain fatty acid (SCFA) concentrations (μmol/g) from ileal, cecal and colonic contents in growing pigs fed a diet with or without a 5% inulin supplement. Total SCFAs are the sum of the following SCFAs: acetate, propionate, isobutyrate, butyrate, isovalerate, valerate. Group differences were tested with Wilcoxon tests. ** *p* < 0.01; * *p* < 0.05. Con, control; Inu, inulin.

As mainly bacterial metabolites, SCFA concentrations in intestinal contents were evaluated to investigate whether the changes in microbial community structure had an impact on its metabolic output. Aligning with the alteration in bacterial communities, the CON and INU pigs had similar SCFA profiles in the ileum and differing in cecum and colon (*p* = 0.03). In the cecum, propionate (*p* = 0.02) and isobutyrate (*p* = 0.01) content was greater while the ratio of acetate to propionate were lower (*p* = 0.02) in the pigs on the inulin diet compared to the control-fed groups. Total SCFA (*p* = 0.04), valerate (*p* = 0.02) and isobutyrate (*p* = 0.01) concentration in colonic contents were significantly increased in the inulin group (Figure 4). Collectively, dietary inulin modulated bacterial community structure in the cecum and colon, and composition differences were associated with different SCFA levels.

### 3.4. Inulin Supplementation Significantly Alters the Plasma Metabolomes

**Figure 5:**
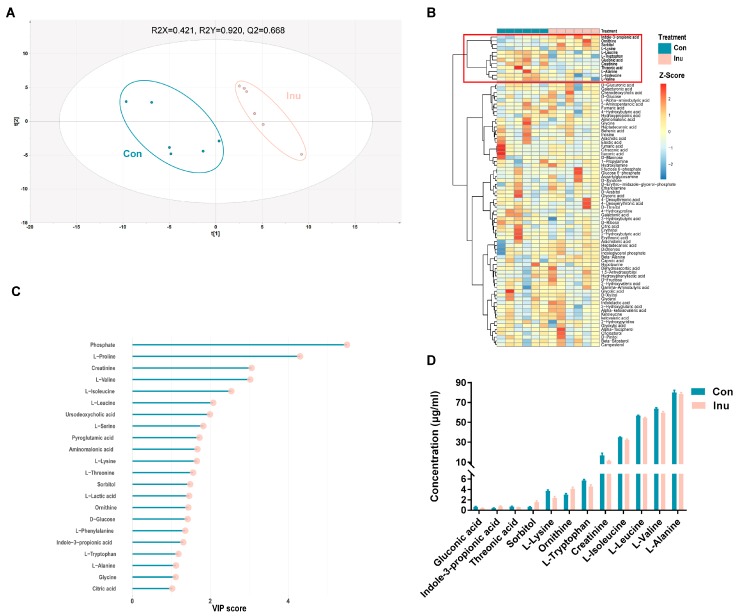
Comparison of plasma co-metabolites in growing pigs fed a diet with or without a 5% inulin supplement (**A**) partial least squares discriminant analysis (PLS-DA) score plot comparing control and inulin. (**B**) Hierarchical clustering result shown as heatmap (distance measure using Euclidean, and clustering algorithm using Ward’s linkage). (**C**) VIP scores estimated from PLS-DA model. Only co-metabolites with a VIP score > 1 were shown. (**D**) Plasma serum co-metabolites which were significantly different between control and inulin. Con, control; Inu, inulin.

A total of 131 plasma metabolites detected using the GC–time-of-flight–MS analytical platform, were annotated in the metabolite database and labeled with the Human Metabolome Database ID (Table S6). A supervised PLS-DA model (*R*_2_ = 0.92, Q_2_ = 0.67, *p* < 0.05) showed robust separation of dietary group, as visualized through the score plot (Figure 5a). A total of 22 metabolites had VIP >1, suggesting that they contribute to the discrimination of the groups (Figure 5b). The metabolites listed were analyzed by multivariate modeling as significant discriminators between treatment groups; 11 metabolites were identified as significant by univariate analysis after FDR correction (Figure 5c). These metabolites, including L-lysine, Indole-Propionic Acid (IPA), L-tryptophan, gluconic acid, ornithine, sorbitol, L-leucine, L-isoleucine, L-valine, threonic acid, L-alanine, and creatinine (Figure 5d). These analyses reveal that there exist distinct plasma metabolites, which could successfully discriminate inulin-feeding conditions from one another.

### 3.5. Microbiota-Metabolites Correlation

**Figure 6:**
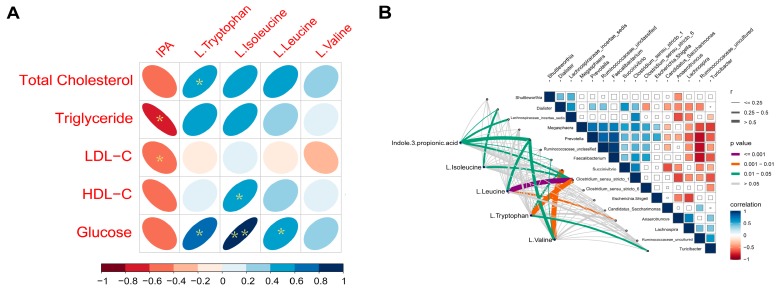
(**A**) Spearman’s correlation matrix of branched-chain amino acids (BCAA), L-trypotophan, indole-3-propionic acid (IPA) and lipid parameters and glucose in growing pigs fed a diet with or without a 5% inulin supplement. The direction of ellipses represents positive or negative correlations and the width of ellipses represents the strength of correlation (narrow ellipse = stronger correlation). IPA, indole-3-propionic acid. * *p* < 0.05; ** *p* < 0.01; (**B**) Pairwise comparisons of cecal genera are shown with a color gradient denoting Spearman’s correlation coefficient. IPA, L-isoleucine, L-leucine, and L-tryptophan was related to each microbial genus by partial Spearman tests. Edge width corresponds to the Partial Spearman’s r statistic for the corresponding distance correlations and edge color denotes the statistical significance.

Spearman correlations were revealed between several plasma AA metabolites and lipid parameters. Positive correlations were found between L-isoleucine (*r* = 0.73, *p* = 0.04), L-leucine (*r* = 0.56, *p* = 0.04), L-tryptophan(*r* = 0.86, *p* < 0.01) and glucose; between L-isoleucine and HDL-C (*r* = 0.63, *p* = 0.03); between L-tryptophan and total cholesterol (*r* = 0.57, *p* = 0.03).Negative correlations were shown between IPA and TG (*r* = −0.81, *p* = 0.02) and LDL-C (*r* = −0.59, *p* = 0.04) (Figure 6a).

Mantel tests were performed to detect correlations between intestinal microbiota and metabolite profiles in the different sites. The ileal microbiota (mantel test, *r* = 0.19, *p* = 0.06) and colonic microbiota (mantel test, *r* = 0.16, *p* = 0.05) didn’t show significant relationship with metabolites profiles, while the cecal microbiota was shown to be positively related with the dissimilarity of metabolites profiles (mantel test, *r* = 0.44, *p* < 0.01). Then, we performed a correlation analysis on the significant cecal microbial taxa, SCFA concentration, and plasma metabolites. The circos plot shows the correlations between gut microbiota, SCFA, and plasma metabolites (Figure S1). Propionate was correlated with *Veillonella* and *Succinivibrio*. Butyrate was associated with an unassigned genus from *Lachnospiraceae* and *Faecalibacterium*. Isobutyrate was positively related to *Prevotella* and negatively related to L-valine. Moreover, valerate had strong associations with *Succinivibrio* and *Clostridium_sensu_stricto_1*.

Interestingly, we found that six genera had a powerful relationship with plasma IPA, they are *Dialister*, *Faecalibacterium*, *Prevotella*, *Lachnospiraceae_incertae_sedis*, *Clostridium_sensu_stricto_6* and *Clostridium_sensu_stricto_1*. Clostridium_sensu_stricto_1 also showed a strong correlation with L-tryptophan, L-isoleucine, and L-leucine. Moreover, we also detected that L-leucine and L-valine had a significant association with *Prevotella*, while *Succinivibrio* was linked with L-tryptophan and L-valine (Figure 6b).

### 3.6. Predicted Function of Microbiota and Pathway Analysis

**Figure 7:**
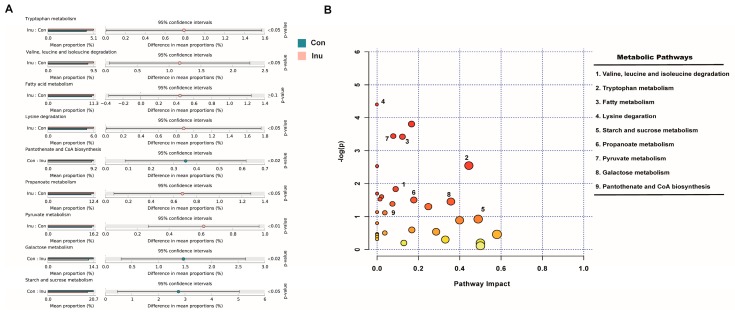
Common functional pathway analysis in growing pigs fed a diet with or without a 5% inulin supplement. (**A**) 16s rDNA sequencing predicted based on SILVA123 database using Tax4Fun; (**B**) significant metabolites predicted based on KEGG database using Metaboanalyst 4.0. The closer to red, the smaller *p* value is.

To better comprehend the functional roles of the cecal microbiome and its metabolites, we used Tax4Fun analysis to predict the functional roles of the cecal microbiota, and MetaboAnalyst 4.0 to investigate metabolic pathways based on the identified plasma metabolites. The result of Tax4Fun analysis showed that 91 functional pathways were significantly altered between inulin and control groups (Table S7). While MetaboAnalyst identified 77 metabolic pathways that might be associated with the changed plasma metabolite profile (Table S8), nine of these metabolic pathways were also found by the Taxt4Fun analysis. To discriminate the potential functions of cecal microbiota in two treatment groups, a two-tail Mann-Whitney U tests was used to the relative abundance of predicted KEGG pathways. Intriguingly, 7 of the nine pathways were upregulated in the inulin group, including fatty acid metabolism, lysine degradation, pantothenate and CoA biosynthesis, propanoate metabolism, pyruvate metabolism, tryptophan metabolism, valine, leucine and isoleucine degradation. In contrast, galactose metabolism and starch and sucrose metabolism were downregulated in the inulin group (Figure 7). Similar results were not obtained in the ileum and colon (Tables S6 and S9). The nine shared pathways predicted by both the cecal microbiome and plasma metabolimics analyses offered solid evidence for cecal microbiome-circulating metabolites axes.

## 4. Discussion

Consumption of inulin has been shown to prevent metabolic syndrome [36], obesity [3], cardiovascular disease [2], and T2D [4]. In the current study, using pigs as an excellent mimic model for human, the regulating effect of inulin on lipid metabolism was confirmed. This study provides a comprehensive view of the changes in circulating metabolites with bioregional microbiota shifts in response to the inulin. These observations offer the notion that the involved and altered metabolic pathways appear associated with the underlying mechanism the influences of inulin on whole-body metabolism.

Microbiota across the ileum, cecum, and colon of pig has been well-characterized by our previous study. The β-diversity was performed by Weighted-Unifrac measurement, which utilizes sequence evolution and abundance to map distance between samples separated by inulin. We observed the clustering of bioregional microbiota between the ileum and hindgut, the influence of inulin on the cecum and colon lumen microbial composition. Although inulin intervention had altered genera in all three gut sites. Mantel test’s results showed that the only connection is between cecal microbial composition and plasma metabolome. These results are in line with a study of inulin degradation in young pigs, which suggests that inulin degrading activity was detectable in digesta from the ileum, cecum, and proximal colon, but the highest activity found in the cecum [37]. Hence, we rationalize that the mechanism by which inulin affects whole-body metabolism si likely driven by cecal bacterial populations, which, in turn, could change specific microbe-derived signaling factors.

Specific bacteria were found to be heavily influenced by inulin across multiple luminal regions. For instance, some well-known fermenters such as *Butyrivibrio* and *Prevotella* are thought to improve intestinal health and have shown an increased level in pigs fed with inulin [38,39,40]. Our results suggest that *Faecalibacterium is* more abundant in cecum and colon of inulin-fed pigs than con-fed pigs. As a traditional target of inulin [18], *Faecalibacterium* is well known for conferring health benefits and has been shown to decrease lipid accumulation, improve insulin sensitivity, and increase fatty acid oxidation and adiponectin signaling [41,42]. Moreover, we found that *Turicibacter* decreased in three gut sites for inulin-fed pigs, an effect which has previously been reported to be correlated with steroid and lipid metabolism [43] and is negatively correlated with protein and energy digestibility in low-fiber feeding growing-finishing pigs [44]. In addition, *Turicibacter* is involved in inhibition of phosphatidylinositol 3-kinase, and triggering c-Jun N-terminal kinase in a rat type 2 diabetic model leads to increased body weight and fasting glucose level [45]. Despite of the correlations that have been established between those intestine microbes and lipid metabolism, the exact metabolic mechanisms remain unclear.

Some of the differences among dietary groups can potentially be attributed to a result of feedback interactions between microbial metabolites and host tissues. The most well-established example of gut-derived regulatory factors is the frequently observed increase in SCFAs in response to inulin intake, which was also consistently observed in our study. The increase of SCFAs was attributed to the inulin-degrading bacteria, which are known to use fermentative metabolisms. *Veillonella* and *Succinivibrio* are propionate-producing bacteria [46,47]. Consistent with our previous study [20], we observed the alteration in propionate concentration and the ratio of acetate to propionate (A:P), which has metabolic regulation. Acetate takes part in de novo lipogenesis, while propionate is considered as a blocker for lipogenesis and can promote insulin resistance [48]. We believe that the decrease in A: P ratio observed in this study, at least, partially contributed to the modulatory effect of inulin on the regulation of lipid metabolism. Besides, we also noted that the concertation of valerate and isobutyrate were elevated in the inulin-fed pigs. In recent studies, valerate was reported to be inversely correlated with the LDL/HDL ratio in the liver of high-fat feeding mice [49], and a valeric acid derivative suppressed cholesterogenesis in rat liver [50]. Surprisingly, we did not find butyrate elevation in this research, but we suspect that butyrate has been quickly absorbed and utilized as energy resource of intestinal epithelial cells and/or bacteria. As major products of valine fermented by gut microflora, isobutyrate could be used as an alternative fuel source when butyrate is scarce [51], and modulate lipid metabolism similarly to SCFAs [52].

The most striking difference in plasma metabolites was the almost universal decrease in several important amino acids. Few studies have shown that the level of circulating amino acids is related to high dietary fiber diets [22,53]. Common to these studies was that some amino acids, including three BCAAs and aromatic amino acids (such as tryptophan), were lower after inulin intervention. These findings were also observed in our study, BCAA had positively related to total cholesterol and glucose, which were reduced in inulin groups. The risk of future T2D was substantially increased with the elevation of a small cluster of circulating amino acids, including BCAAs and aromatic amino acids [54]. As the activation of the mammalian target of rapamycin (mTOR) and its downstream effectors, S6 kinase and insulin receptor substrate-1, the elevating BCAA may disrupt insulin signaling, thus triggering T2D [54,55]. Interestingly, many research and clinical trials have supplied significant and accordant evidence that inulin can reduce T2D risk and improve glycemic control in people or rodents with T2D [56,57]. Beyond BCAA, we identified a variety of candidate xeno-metabolites that are altered by changes in the gut bacteria and that reach the systemic circulation. For instance, as a kind of microbiome-associated catabolites of tryptophan, IPA had increased along with tryptophan reduced in plasma of the inulin group. IPA has a hypoglycemic function [58], which is considered to protect against T2D via activating peroxisome proliferator-activated receptor (PPAR) subtypes α and γ [59]. Taken together, we speculate that the alteration of several circulating amino acids and its microbe-derived catabolites may be one of the mechanisms through which the inulin exerts its beneficial effects on T2D.

The change in blood amino acid concentrations is contributed to complicated interactions among dietary intake, tissue breakdown, de novo synthesis, and other factors such as gut microbial activity [60,61]. Many studies have shown that dietary fiber might affect amino acid metabolism. In a randomized control trial, high-fiber rye bread intake had reduced serum BCAA levels significantly [62]. Moreover, the ingestion of resistant starch leads to a 22-fold increase in plasma IPA [63]. Dietary fiber could regulate amino acid metabolism in gut microbiota either by affecting the microbial composition and abundance or by providing the carbon source for bacterial growth [64]. Multi-omics analysis identified several genera associated with inulin intake, and some of these genera were correlated with plasma BCAA, tryptophan, and IPA concentrations. A recent study reported that high dietary fiber enables overgrowth of *Prevotella* in vegetarians; this genus was also negatively associated with circulating BCAA concentration [65]. This evidence was also observed in the present study, where the *Prevotella* was higher in the inulin groups and had a negative correlation with L-isoleucine and L-valine. Such an increase in this genus has been validated in the dietary-fiber-induced melioration in glucose metabolism [66]. The genera *Dialister* and *Clostridium_sensu_stricto_1* were closely positively correlated with circulating plasma IPA. *Clostridium_sensu_stricto_1* and other *Clostridia* species, which metabolize tryptophan into IPA [67], have been shown to boost in our study and also in other fiber-rich diets [68]. *Dialister* was previously shown to be one of the amino acid-metabolizing bacteria. Its increased abundance in the microbiota of healthy adult men consuming soluble corn fiber [69]. This genus was also positively related to indole production and could be stimulated by mannooligosaccharides in vitro [70,71]. Except for microbial composition, predicated function analysis in the present study detected that the two pathways of the gut microbial community, namely valine, leucine, and isoleucine degradation and tryptophan metabolism, were upregulated in the inulin group compared with the control group. The upregulated gut microbial BCAAs degradation pathway and tryptophan metabolism in the combined inulin group could also help to explain the lower concentrations of circulating BCAAs and the higher concentrations of IPA in inulin-fed pigs. Future researches are warranted to validate this finding.

## 5. Conclusions

In summary, our study showed and illustrated an evident alteration in diversity and several genera in the different gut regions after inulin consumption, with more significant differences observed in the cecum and colon. Plasma metabolomics analysis revealed that BCAAs, tryptophan, and IPA featured in inulin-fed pigs only correlated with gut bacteria in cecum, suggesting that the cecum was the primary region for host–microbe interplay upon inulin intervention. Our study further supports Dutch microbiologist Lourens Baas Becking’s hypothesis, “everything is everywhere, but the environment selects” [71]. Concerning the limitations of the current study, the sample size of the present study was relatively small. However, we are confident about our finding because gut microbiota alteration and plasma metabolomic results are similar to those observed in previous works. More detailed studies regarding the intestinal production of xeno-metabolites, such as BCAAs, IPA, and their translocation to the circulation, should be carried out. Nonetheless, our data give comprehensive insights into the host–microbe interplay in the intestine of swine, furthering our knowledge in enhancing pigs and human health via dietary intervention.

## Supplementary Materials

The following are available online at https://www.mdpi.com/2076-2607/8/1/111/s1, Figure S1: Circos plot showing the significant correlations between microbial, SCFAs, and metabolomics data. A correlation cut off of 0.60 was considered significant. Positive correlation is shown in red, and negative correlation is shown in blue line. Table S1: Composition of diets. Table S2; Effects of inulin on the apparent total intestinal digestibility of nutrients in growing pigs. Table S3: Alpha-diversity of ileum, cecum, and colon gut microbiota from pigs fed inulin. Table S4: Alpha-diversity of ileum, cecum, and colon gut microbiota from pigs fed inulin. Table S5: Genera altered by diet in ileum, cecum, and colon of the growing pigs provided inulin. Table S6: Qualitative and quantitative analysis of plasma metabolites profiling of the control and inulin groups. Table S7: Functional pathways predicted by Tax4Fun analyses altered by diet in cecum of growing pigs provided inulin. Table S8: Functional pathways predicted by Tax4Fun analyses altered by diet in ileum of growing pigs provided inulin. Table S9: Functional pathways predicted by Tax4Fun analyses altered by diet in colon of growing pigs provided inulin.
